# Revisiting the window of opportunity for cotranscriptional splicing in budding yeast

**DOI:** 10.1261/rna.075895.120

**Published:** 2020-09

**Authors:** Vahid Aslanzadeh, Jean D. Beggs

**Affiliations:** Wellcome Centre for Cell Biology, University of Edinburgh, Edinburgh EH9 3BF, United Kingdom

**Keywords:** introns, RNA polymerase speed, RNA polymerase, yeast

## Abstract

We reported previously that, in budding yeast, transcription rate affects both the efficiency and fidelity of pre-mRNA splicing, especially of ribosomal protein transcripts. Here, we report that the majority of ribosomal protein transcripts with non-consensus 5′ splice sites are spliced less efficiently when transcription is faster, and more efficiently with slower transcription. These results support the “window of opportunity” model, and we suggest a possible mechanism to explain these findings.

## INTRODUCTION

### Cotranscriptional splicing

The coding sequences of most eukaryotic genes are interrupted by introns (mainly non-coding sequences) that are also present in the nascent transcripts. Pre-mRNA splicing is the process that removes the introns and joins the flanking sequences (exons) to produce mature mRNAs. Evidence accumulating during the last two decades suggests that many, and possibly the majority of, splicing events occur cotranscriptionally, that is before transcription termination (Ameur et al. 2011; Khodor et al. 2011; Carrillo Oesterreich et al. 2016). This raises the intriguing possibility of functionally significant interactions between splicing, chromatin, transcription and other RNA processing events, when they occur in close proximity (Li and Manley 2006; Aitken et al. 2011; Neugebauer 2019). Two models were proposed to explain how transcription and splicing are coupled, referred to as the recruitment coupling and the kinetic coupling models, which are not mutually exclusive (for review, see Dujardin et al. 2013).

### Recruitment coupling

The term “recruitment coupling” refers to the ability of the transcription machinery to promote the recruitment of RNA processing factors to the site of transcription. In particular, the carboxyterminal domain (CTD) of the largest subunit of RNA polymerase II (RNAPII) acts as a “landing pad” for cotranscriptional recruitment of capping, splicing and 3′ end processing factors to nascent RNA (Bentley 2014). Strong support for this model was obtained by a study with human cell lines showing CTD-dependent inhibitory action by serine/arginine-rich (SR) splicing factor SRSF3 (SRp20) in inclusion of fibronectin cassette exon 33 (E33) (de la Mata and Kornblihtt 2006).

### Kinetic coupling

Early evidence for coupling between transcription and splicing was reported by Eperon et al. (1988), who showed that, in HeLa cells, the use of an alternative splice site positioned within a potential stem–loop structure depended on a “window” of availability of the splice site in the nascent RNA for splicing factors or hnRNP proteins to bind before the inhibitory stem–loop formed. A prediction of their model was that the rate of transcription elongation could control alternative splicing by determining the duration of the window. Indeed, de la Mata et al. (2003) showed that, in cultured human cells, a slow RNAPII promoted inclusion of the fibronectin EDI (extra domain I) exon, and they proposed that slower transcription elongation expanded the “window of opportunity” for recognition of the weak 3′ splice site (3′SS) upstream of the exon before transcription of a competing 3′SS downstream. In this model, the rate of transcript elongation could affect the extent and even the order in which splicing factors assemble cotranscriptionally on a nascent transcript, although it need not affect splicing catalysis, which may occur post-transcriptionally on cotranscriptionally assembled spliceosomes.

Evidence of kinetic coupling was obtained also in budding yeast by Howe et al. (2003), who observed enhanced inclusion of the second exon of modified *DYN2* transcripts in a slow RNAPII mutant or when cells were treated with chemical inhibitors of transcription. In striking contrast, slower elongation caused skipping of human CFTR exon 9 in a minigene construct due to enhanced recruitment of ETR-3, a negative splicing factor, to the 3′SS of the exon (Dujardin et al. 2014). This demonstrates that slower transcription can also expand the window of opportunity for recruitment of factors that block splicing of a newly transcribed exon, reducing its inclusion rate. Overall, it seems that transcription rate determines the temporal window of opportunity for selection or rejection of an upstream sequence before a competing downstream sequence is transcribed. However, Fong et al. (2014) found that, in human cells, both faster and slower elongating RNA polymerase mutants may disrupt splicing in the same way, which seems contrary to the “window of opportunity” model. They proposed that an optimal rate of transcriptional elongation is required for normal cotranscriptional pre-mRNA splicing. Therefore, for the vast majority of genes, it is unclear what determines the splicing outcome of altering transcription elongation rate or how changes in transcription rate are regulated locally at alternatively spliced exons. It has been proposed that mechanisms may exist to slow or pause transcription downstream from introns in budding yeast, thereby stretching the window of opportunity for cotranscriptional spliceosome assembly to occur (Alexander et al. 2010; Oesterreich et al. 2010).

### Transcription rate affects splicing efficiency

To investigate the effect of transcription elongation rate on splicing efficiency (the fraction of transcripts that get spliced) in budding yeast (Aslanzadeh et al. 2018), we previously used RNAPII trigger loop mutants that elongate, on average, four times faster (Rpb1-*G1097D*) or eight times slower (Rpb1-*H1085Y*) than wild-type RNAPII (∼12 nt) in vitro (Kaplan et al. 2012). In all these strains, *UPF1* was deleted to reduce degradation caused by nonsense mediated decay. Both 4-thio-uracil labeling for analysis of newly synthesized RNA and Native Elongating Transcript (NET)-RTqPCR to analyze RNAPII-associated transcripts for several genes showed that more splicing occurs cotranscriptionally with the slow mutant, and less with the fast mutant compared with wild-type RNAPII. Importantly, these differences were not simply due to there being more or less unspliced pre-mRNA as a consequence of different decay rates in the mutants, as the level of spliced mRNA was elevated in the slow mutant and reduced in the fast mutant compared with the WT. These results are compatible with both recruitment coupling and kinetic coupling; slower elongation allows more time for cotranscriptional recruitment of splicing factors to the nascent transcript, whereas faster elongation has the opposite effect. Moreover, analysis of total RNA (as opposed to nascent RNA analyzed by NET-RTqPCR) revealed that overall splicing efficiency was also decreased with the fast RNAPII mutant and slightly improved with the slow mutant compared to wild-type. These results indicate that post-transcriptional splicing in the fast mutant does not compensate for its reduced cotranscriptional splicing (Aslanzadeh et al. 2018).

Interestingly, transcriptome-wide analysis of splicing efficiency by RNA sequencing of total RNA revealed that the effect of the fast RNAPII mutant was mainly to reduce splicing efficiency with ribosomal protein (RP) coding transcripts (seen as increased accumulation of unspliced pre-mRNAs; Fig. 1C in Aslanzadeh et al. 2018), whereas the slow mutant increased the splicing efficiency with both RP and non-RP transcripts.

**FIGURE 1.**
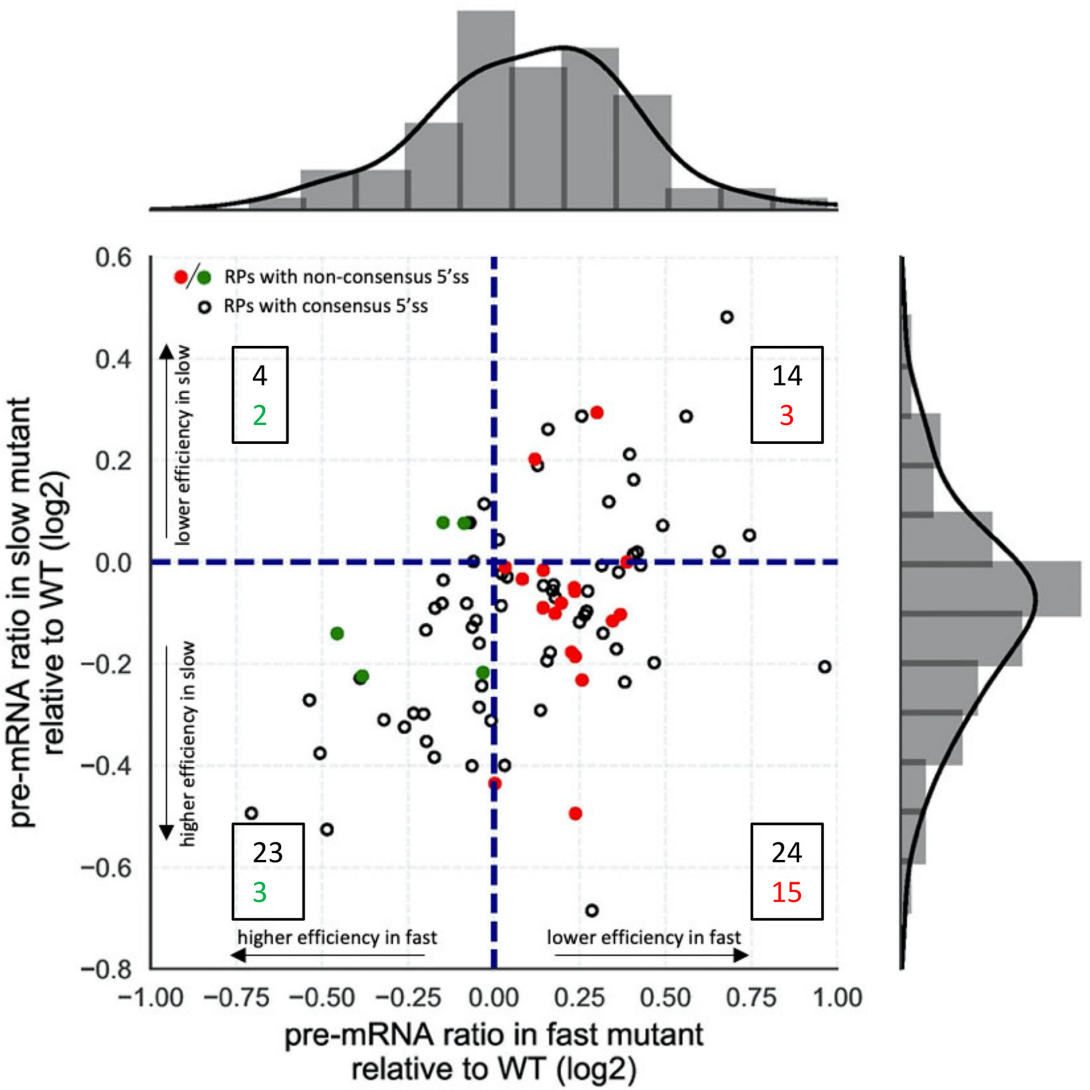
Faster transcription reduces and slower transcription increases the efficiency of splicing RP transcripts with non-canonical 5′ splice sites. Splicing efficiencies were calculated (Supplemental Table S1) as RNA-seq read counts from pre-mRNA divided by total reads (pre-mRNA + mRNA) for each transcript (Aslanzadeh et al. 2018). Transcripts that splice significantly more or less efficiently in the fast mutant compared to WT lie to the *left* and *right* of the vertical dashed line, respectively. Those that splice more or less efficiently in the slow mutant compared to WT lie *below* or *above* the horizontal dashed line, respectively. RP transcripts with non-consensus 5′SS are represented by green (more efficiently spliced in the fast mutant) or red (less efficiently spliced in the fast mutant) dots. The boxed numbers indicate the number of genes represented in each quadrant, consensus in black, non-consensus in colored font. Histograms show the distribution of pre-mRNA ratios relative to WT in the fast (*top*) and slow (*right*) mutants. In the fast mutant there are more genes with increased pre-mRNA ratio (reduced splicing efficiency) relative to WT and in the slow mutant there are more genes with reduced pre-mRNA ratio (increased splicing efficiency) relative to WT.

We previously reported that RP transcripts are spliced faster and more cotranscriptionally (Barrass et al. 2015; Wallace and Beggs 2017) and that they tend to be spliced with greater efficiency and higher fidelity (Aslanzadeh et al. 2018). Taken together with the greater sensitivity to transcription speed, these observations indicate that the splicing of RP transcripts is more functionally coupled to transcription than that of non-RP transcripts, and that this coupling is highly beneficial. Yet, it remains largely unknown what determines the extent to which splicing of a transcript is more or less coupled to transcription. Notably, many RP transcripts are spliced more efficiently with slower transcription and less efficiently with faster transcription. Here, we investigate whether this can be explained by variation in splice site sequences.

## RESULTS AND DISCUSSION

Figure 1 shows the effects of the fast and slow RNAPII mutants on RP splicing efficiencies plotted relative to wild-type (which normalizes for effects of expression level in the data analysis) for 88 RP transcripts, using data (Supplemental Table S1) from Aslanzadeh et al. (2018). For 65 RP introns (74% of the 88 RP introns analyzed) splicing efficiencies are higher in the slow RNAPII mutant compared to wild-type (Fig. 1, all dots below the horizontal dashed line) and for 56 RP introns (64%) splicing efficiencies are lower in the fast mutant (all dots right of the vertical dashed line). The quadrant containing data from the largest number of genes is bottom right, with 39 introns (44% of the 88 RP introns analyzed) whose splicing efficiency is both reduced with the fast RNAPII mutant and increased with the slow mutant. Therefore, for 44% of the RP genes the effect of RNAPII elongation rate on efficiency of splicing fits the window of opportunity model.

It was noted that 23 of the 88 RP transcripts have non-consensus 5′ splice sites (5′SS) (i.e., do not match the consensus sequence GUAUGU), and that 18 (78%) of these were spliced more efficiently in the slow strain (colored dots below the horizontal dashed line in Fig. 1; Supplemental Table S2). The same number (18) with non-consensus 5′SS were spliced less efficiently in the fast strain (red dots right of the vertical dashed line in Fig. 1). Conceivably, the reduced splicing efficiency observed for these RP transcripts in the fast mutant could be explained by a combination of delayed splicing due to the non-consensus 5′SS and the shorter window of opportunity for splicing to occur cotranscriptionally due to the faster transcription elongation, but which is compensated by slow transcription. Consistent with this explanation, 15 of these introns with non-consensus 5′SS, splicing was both less efficient in the fast RNAPII strain and more efficiently in the slow strain (red dots in the bottom right quadrant in Fig. 1; Supplemental Table S1). Therefore, the splicing efficiencies for 65% of the RP transcripts with non-consensus 5′SS fit the window of opportunity model. This compares with 24 of 65 introns (37%) with consensus 5′SS that fit this model, which is itself notable (expect ∼25% if the effect of transcription speed was random). It appears, therefore, that the splicing of RP introns with non-consensus 5′SS is more highly coupled to transcription.

Interestingly, genome-wide studies in mammalian cells also found that specific transcript features, such as suboptimal splice sites, may make a particular splicing event more sensitive to transcription rate, especially in transcripts that encode RNA binding proteins, such as ribosomal proteins, or RNA processing factors (Ip et al. 2011; Fong et al. 2014).

For five RP introns with non-consensus 5′SS (*RPS14B*, *RPS19A*, *RPS21B*, *RPL30*, and *RPL43B*) splicing was more efficient rather than less efficient in the fast RNAPII strain (green dots in Fig. 1), suggesting that, for these transcripts, other factors have a greater influence on splicing efficiency than 5′SS. In the case of two of these transcripts, *RPS14B* and *RPL30*, their protein products Rps14p and Rpl30p, when in excess, bind to their respective precursor transcripts and inhibit splicing (Fewell and Woolford 1999; Macías et al. 2008). Therefore, in these two cases at least, competition between cotranscriptional spliceosome assembly and feedback inhibition of splicing by the protein product, may explain the different response to changes in transcription speed, for example if faster transcription through the intron permits spliceosome assembly or optimal secondary structure in the transcript before binding of the inhibitory protein. It would be interesting to investigate whether splicing of the other three transcripts in this category is also subject to negative regulation.

Curiously, the same trends are not apparent among non-RP transcripts with non-consensus 5′SS, but many of these have other atypical intron features that may affect their splicing kinetics.

**FIGURE 2.**
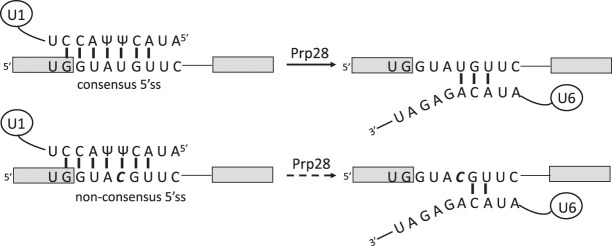
A potential mechanism to explain the observed effects of transcription speed with the suboptical 5′ splice site GUACGU. Gray boxes, exons. (*Upper*) Base-pairing of a canonical 5′SS with U1 snRNA, and with U6 snRNA following Prp28 function. (*Lower*) Likely base-pairing of the non-canonical 5′SS, GUACGU, with U1 snRNA, and with U6 snRNA following Prp28 function. Vertical bars do not indicate the strength of base-pairing.

Our observation that a non-consensus 5′SS correlates with sensitivity to transcription elongation rate supports both the recruitment and kinetic coupling models and suggests that, at least for RP transcripts, a non-consensus 5′SS may reduce or slow spliceosome assembly and/or splicing catalysis in a manner that is compensated by slower transcription elongation. The most frequently occurring non-consensus 5′SS in the RP genes has the sequence GUACGU (Supplemental Table S2). Carrillo Oesterreich et al. (2016) showed, using a reporter gene, that this sequence delayed spliceosome assembly and cotranscriptional splicing compared with a consensus 5′SS, which they proposed could be due to weakened 5′SS base-pairing with U1 snRNA delaying spliceosome assembly. However, it is questionable whether replacing U with C as the base at position +4 of a 5′SS significantly alters the stability of the 5′SS:U1 snRNA interaction. The opposing base at position +5 in U1 snRNA is pseudoU (Ψ) (Plaschka et al. 2018), which can pair weakly with either U or C, with relative stabilities depending on the context of the surrounding sequence (Kierzek et al. 2014) and contacting proteins. Interestingly, in the study by Carrillo Oesterreich et al. (2016), ChIP analysis showed U1 snRNP apparently recruited equally efficiently to reporter transcripts containing either GUAUGU or GUACGU at the 5′SS, whereas the U1 snRNP ChIP signal was lost more slowly from the GUACGU reporter. A possible explanation for this delayed departure of U1 snRNP from the cotranscriptionally assembling spliceosomes is the reduced ability of CGU in the non-consensus 5′SS to pair with ACA in the ACAGAGA motif of U6 snRNA, an interaction that is coupled with U1 snRNP displacement from the 5′SS by Prp28 (Fig. 2; Staley and Guthrie 1999; Chen et al. 2001). In this case, slower transcription elongation would allow more time for the assembling spliceosome to proceed through this checkpoint cotranscriptionally and/or for cotranscriptional recruitment of factors required to form catalytically active spliceosomes, providing a rational explanation for the effect of transcription speed on splicing efficiency for this subset of transcripts.

Overall, the work discussed here supports the window of opportunity model as an explanation for the effects of transcription speed on splicing efficiency of many RP transcripts, especially those with non-consensus 5′SS. We propose a possible mechanistic explanation for this as being mediated by effects on cotranscriptional spliceosome assembly. For a small number of RP introns feedback regulation by their gene products may explain why splicing is more efficient with the faster RNAPII. The work by Fong et al. (2014) challenged the window of opportunity model, finding that for many human genes both faster and slower elongating RNAPII mutants seemed to disrupt splicing in the same way. However, whereas their slow mutant elongated 3.4 times slower than normal RNAPII, their fast RNAPII mutant elongated only 12% faster than normal, resulting in quantitatively smaller changes in splicing, which may not be very revealing. In comparison, the yeast mutants used in this study elongate on average eight times slower or four times faster than wild-type RNAPII (Kaplan et al. 2012), which may provide a more robust comparison. Perhaps more importantly, Fong et al. measured changes in alternative splicing events, whereas this yeast study monitored changes in splicing efficiency. Conceivably, the effects of transcription speed on alternative splicing may be more complex.

What other features of RP transcripts might cause their splicing to be more coupled to transcription? Promoter structure was shown to be important for alternative splicing in human cells (Cramer et al. 1997) and yeast (Kawashima et al. 2014), and it was shown that RP transcripts in budding yeast have distinct promoter architectures, with an exclusive pattern of DNA binding proteins that enhance their transcription (Knight et al. 2014). Indeed, considering that RP genes are more highly expressed than most non-RP genes, expression level could be an underlying factor in determining the extent of coupling. Chromatin structure and modifications also affect both transcription and splicing (Gunderson et al. 2011; Neves et al. 2017; Venkataramanan et al. 2017). One informative future approach would be to study the effect on cotranscriptional splicing of swapping the promoters and different intron features of yeast RP and non-RP transcripts.

## MATERIALS AND METHODS

For details of the data generation and analysis methods, see Aslanzadeh et al. (2018).

## SUPPLEMENTAL MATERIAL

Supplemental material is available for this article.

## Supplementary Material

Supplemental Material
