# Supplementary material for: Revisiting the window of opportunity for cotranscriptional splicing in budding yeast
Source: RNA. 2020 Sep;26(9):1081–5. doi: 10.1261/rna.075895.120 (PMC7430680; doi:10.1261/rna.075895.120)
Supplement: Supplemental Material [file supp_26_9_1081__index.html]

Revisiting the window of opportunity for co-transcriptional splicing in budding yeast — Revisiting the window of opportunity for cotranscriptional splicing in budding yeast — Supplemental Material 

# Revisiting the window of opportunity for cotranscriptional splicing in budding yeast

## Supplemental Material

- Supplemental\_Tables.pdf
