# Supplementary material for: Revisiting the window of opportunity for cotranscriptional splicing in budding yeast
Source: RNA. 2020 Sep;26(9):1081–5. doi: 10.1261/rna.075895.120 (PMC7430680; doi:10.1261/rna.075895.120)
Supplement: Supplemental Material [file supp_075895.120_Supplemental_Tables.pdf]

Revisiting the window of opportunity for co-transcriptional splicing efficiency in budding yeast

Vahid Aslanzadeh[1,2] and Jean D. Beggs[1*]

Supplemental Information

Table S1

RNA-seq read counts for pre-mRNAs in the fast RNAPII mutant (columns A,B show counts for 2 biological replicates), slow mutant (C,D), wild-type (WT; E,F) or for spliced mRNAs (H-M). Pre-mRNA read counts calculated as sum of the reads aligning to 5'SS, intron, 3'SS and the reads spanning on exon1-intron-exon2 sequence. mRNA read counts calculated as sum of the reads aligning to exon1 – exon2 splice junction. Pre-mRNA ratios for fast, slow and WT (columns N,O,P respectively were calculated as counts of pre-mRNA divided by total read counts (pre-mRNA+mRNA)); log2 ratio of mutant/WT for fast (S) and slow (T) RNAPII mutants. The second sheet (named 'RP') shows the same data for RP transcripts only, indicating those with non-consensus 5'SS that splice more efficiently (green) or less efficiently (red) in the fast RNAPII mutant.

Table S2

Transcripts with non-consensus 5'SS whose splicing efficiency was measurable in RNA-seq data from (Aslanzadeh et al., 2018), indicating those with improved (green) or reduced (red) splicing efficiency in the fast mutant compared to WT. The list of genes with non-consensus 5'SS sequences was obtained using the search facility of the Ares Lab Yeast Intron Database: http:// intron.ucsc.edu/yeast4.3/, and checked in the Saccharomyces Genome Database: https:// www.yeastgenome.org/

Table S1

	premRNA_fast_A	premRNA_fast_B	premRNA_slow_A	premRNA_slow_B	premRNA_wt_A	premRNA_wt_B	mRNA_fast_A	mRNA_fast_B	mRNA_slow_A	mRNA_slow_B	mRNA_wt_A	mRNA_wt_B	premRNA ratio_fast	premRNA ratio_slow	premRNA ratio_wt	premRNAratio_fast/wt (log2)	premRNA ratio_slow/wt(log2)
YLR445W	22	63	38	61	17	22	0	30	2	9	25	5	0.83871	0.910714	0.609788	0.459863	0.57869
RPS21A	2681	2194	1338	2088	2601	2657	31967	32994	19657	27747	29812	27827	0.069865	0.066857	0.083703	-0.26072	-0.3242
RPS21B	886	935	744	1058	1233	1217	28754	29480	20819	30790	31625	28061	0.030317	0.033862	0.039546	-0.38342	-0.22387
YBL059W	340	526	291	445	298	371	646	442	390	631	243	272	0.444108	0.420441	0.563907	-0.34455	-0.42356
RAD14	86	150	135	110	47	55	246	289	300	294	187	225	0.300361	0.291311	0.198642	0.596529	0.552392
COF1	922	1075	644	1268	958	1017	5859	5744	6647	9631	6876	6690	0.146808	0.102335	0.127123	0.207708	-0.31293
YBR090C	1265	1073	1472	1733	1638	1438	2033	1762	2070	2534	1831	1523	0.381025	0.410862	0.478914	-0.32988	-0.22111
LSB3	197	172	142	238	292	213	1737	1439	2112	2254	1729	1546	0.104314	0.079252	0.132787	-0.34819	-0.74459
RPS11B	2947	3426	1945	2912	3469	2996	23216	21589	19059	23283	29458	25085	0.124799	0.101884	0.106023	0.235231	-0.05745
RPS11A	2284	2349	1378	2189	2451	2033	44844	40867	41816	49592	60786	47667	0.051409	0.037088	0.039832	0.368095	-0.10297
YDR261W-	13	10	1	87	13	9	211	165	203	318	128	136	0.057589	0.109858	0.077134	-0.42156	0.51021
YIL156W-B	347	214	429	621	322	376	599	860	777	1057	846	965	0.283031	0.362902	0.278036	0.025688	0.384308
IWR1	725	1321	967	1737	1383	1288	311	372	351	381	124	244	0.740039	0.7769	0.879224	-0.24863	-0.1785
CIN2	776	750	532	808	724	556	206	280	197	176	218	205	0.75919	0.775453	0.749598	0.018344	0.048922
NCB2	163	106	112	243	255	209	404	484	530	597	310	431	0.233569	0.23187	0.388945	-0.73571	-0.74625
HRB1	1049	1507	1359	2059	1902	1896	447	448	614	702	563	371	0.736024	0.717272	0.803975	-0.1274	-0.16463
GPI15	111	149	167	228	189	191	325	569	495	801	595	460	0.231054	0.23692	0.267233	-0.20987	-0.1737
MOB1	151	174	242	386	238	201	251	305	262	461	232	271	0.369439	0.467942	0.466115	-0.33535	0.005644
CPT1	51	71	42	152	115	81	1334	1662	1295	1843	1484	1566	0.038896	0.053802	0.06055	-0.6385	-0.17047
RPS19B	2845	2911	1683	2603	2719	2406	25124	25245	22186	28165	27950	25344	0.102554	0.077555	0.08768	0.226072	-0.17701
RPS19A	2397	2393	1629	2372	2452	2225	16794	16373	13905	17881	15652	15929	0.12621	0.110993	0.129001	-0.03156	-0.21692
BOS1	246	293	220	185	211	268	225	223	272	389	224	255	0.545061	0.384727	0.498743	0.128122	-0.37446
YLR464W	405	506	369	533	472	428	0	0	0	0	0	0	1	1	1	0	0
ACT1	797	591	623	1124	762	888	9313	9012	9679	14803	12787	11723	0.070188	0.065523	0.063328	0.148393	0.049165
RPS6B	4319	4714	2009	3067	3773	3055	13989	16458	11840	18193	17903	16307	0.22928	0.144663	0.165923	0.466594	-0.19782
RPS6A	1627	1788	1264	2138	1999	1861	6254	6308	6391	9254	8148	8710	0.213648	0.176398	0.186526	0.195859	-0.08054
RPO26	388	518	656	800	774	761	1785	1731	2349	2777	2945	2348	0.20444	0.220977	0.226447	-0.1475	-0.03528
MEI4	44	119	54	99	91	193	0	0	10	0	0	0	1	0.921875	1	0	-0.11736
DMC1	547	662	268	597	375	374	4	32	0	42	47	8	0.973315	0.967136	0.933842	0.05973	0.050541
APE2	3865	3726	3326	5183	3850	3673	535	639	440	541	419	381	0.866009	0.894325	0.903935	-0.06184	-0.01542
SPT14	448	529	308	381	522	290	55	71	31	34	58	52	0.886161	0.913313	0.873977	0.019975	0.063515
RPS22B	4582	4571	2017	3779	5328	5360	14320	13836	8298	9625	11326	9231	0.245369	0.238736	0.343636	-0.48593	-0.52547
RPL42B	2253	2601	1548	2454	2372	2549	14555	13001	8923	11540	16536	15128	0.150376	0.161599	0.134824	0.157499	0.261339
TDA5	122	171	135	199	367	305	404	354	447	521	297	260	0.278827	0.254174	0.546267	-0.97024	-1.10379
KIN28	425	330	508	670	460	374	297	232	228	247	169	189	0.587916	0.71043	0.697809	-0.24722	0.025861
BET1	113	235	173	398	250	246	448	563	532	1001	676	518	0.247956	0.264939	0.295984	-0.25543	-0.15986
ARP9	82	92	139	333	137	107	557	528	719	736	461	487	0.138356	0.236755	0.204616	-0.56453	0.210479
RPS10A	2958	3108	2017	3432	3742	3886	39842	37670	35960	43875	45573	40250	0.072665	0.062829	0.081963	-0.17371	-0.38353
RPS10B	2235	2413	1848	2647	2051	2076	30938	27425	24684	28147	24466	21717	0.074122	0.077805	0.0823	-0.15098	-0.08102
TMA20	286	360	160	412	313	254	830	1032	1250	1677	1066	1376	0.257447	0.155349	0.191402	0.427666	-0.30109
YDR535C	643	465	354	315	175	289	0	0	0	0	0	0	1	1	1	0	0

	premRNA_ fast_A	premRNA_ fast_B	premRNA_ slow_A	premRNA_ slow_B	premRNA_ wt_A	premRNA_ wt_B	mRNA_ fast_A	mRNA_ fast_B	mRNA_ slow_A	mRNA_ slow_B	mRNA_ wt_A	mRNA_ wt_B	premRNA ratio_fast	premRNA ratio_slow	premRNA ratio_wt	premRNAra tio_fast/wt (log2)	premRNA ratio_slow/ wt(log2)
RPL42A	11151	11837	12080	16110	16205	16179	5470	4413	4676	5711	6610	5455	0.699665	0.729608	0.729064	-0.05938	0.001075
ARP2	226	208	208	318	243	205	1577	1737	2277	3003	2128	1711	0.116144	0.089728	0.104741	0.149084	-0.22319
UBC8	118	126	168	173	104	82	153	184	164	361	111	158	0.420938	0.414997	0.412694	0.028536	0.008029
UBC9	200	203	190	270	217	275	1324	1333	1019	1496	1456	1286	0.131698	0.155021	0.152938	-0.21572	0.019518
HPC2	29	111	47	136	94	95	183	147	187	222	103	150	0.283513	0.290371	0.432456	-0.60914	-0.57465
MAF1	115	129	87	146	114	163	502	558	276	543	386	366	0.187079	0.225785	0.268064	-0.51893	-0.24763
YRA1	6291	6815	4152	6033	3628	3337	3977	3961	2845	3630	2571	2972	0.622552	0.608869	0.557091	0.160281	0.128217
ASC1	4257	4757	2087	3579	3332	3246	34037	38177	21126	34035	33712	36569	0.110982	0.092529	0.085737	0.372336	0.109981
RPS18A	2935	2986	2103	3215	3425	3251	29420	29306	23611	33687	30949	33262	0.091591	0.084453	0.094338	-0.04264	-0.15968
RPS18B	1113	1204	971	1151	1045	1252	23529	24150	21514	31723	30896	32434	0.046327	0.039098	0.034942	0.40691	0.162161
PBA1	96	153	99	193	147	150	187	286	288	351	211	232	0.343871	0.305297	0.401642	-0.22404	-0.3957
YLR211C	12	11	34	84	37	27	34	86	102	129	47	38	0.187136	0.322183	0.42793	-1.19329	-0.4095
MRPL44	233	270	418	595	421	334	1146	1178	2220	2613	760	1017	0.177714	0.171964	0.301851	-0.76428	-0.81173
RPL14A	4015	4266	3099	3925	4950	4539	70593	66837	46404	65645	70143	65670	0.056906	0.05951	0.065284	-0.19815	-0.1336
RPL31A	2001	2007	1469	2027	1901	1935	61377	58478	41713	51267	58439	50652	0.032377	0.036027	0.03415	-0.07693	0.077154
RPL31B	947	1042	993	1240	1704	1264	9305	9366	11632	15559	13204	13276	0.096244	0.076234	0.100617	-0.06411	-0.40037
RPL14B	2369	2245	1641	2333	2949	2462	24791	23821	14367	19682	18894	18656	0.086676	0.104242	0.125796	-0.53739	-0.27115
ERD2	205	146	148	211	120	94	454	488	394	862	558	444	0.270681	0.234854	0.175856	0.622196	0.417367
RPL37B	2912	3477	1721	2196	1860	1567	13952	11459	8860	10506	11712	9786	0.202734	0.167768	0.137536	0.559781	0.286659
RPL16B	1573	1746	1120	1453	1863	1484	28001	27449	26926	33026	36431	35372	0.056497	0.041038	0.044457	0.345744	-0.11546
RPL16A	2026	2146	1419	2041	2529	2198	17526	16226	17937	22324	24017	22443	0.110215	0.078539	0.092235	0.256934	-0.2319
RPL37A	3353	3119	2428	3464	3589	3342	7605	7058	6854	7806	8238	7791	0.306231	0.284473	0.301823	0.020915	-0.08541
RPS17A	8545	7274	6069	7438	9703	8882	17861	17134	15351	20995	26661	21157	0.310809	0.272465	0.281256	0.144144	-0.04581
RFA2	134	192	133	208	170	124	320	333	233	390	374	298	0.330434	0.355607	0.303169	0.124239	0.23016
COX5B	1061	1121	631	903	528	630	409	409	857	1384	451	352	0.727224	0.40945	0.590437	0.300617	-0.5281
RPS17B	2307	2659	1159	1809	2513	2020	4141	4492	4621	5765	5737	4407	0.364811	0.219681	0.309453	0.23743	-0.49431
RPS27B	1964	2719	1359	2193	2391	1937	16178	15957	15716	23960	24736	24605	0.126923	0.081721	0.08056	0.655815	0.020653
RPL27B	2098	2648	1497	2016	2164	2386	8300	8720	7633	10549	10009	10097	0.217352	0.162205	0.184455	0.236763	-0.18545
RPL27A	3333	3037	2196	3149	2675	2548	32790	33478	24217	31250	34002	34096	0.08772	0.087342	0.071234	0.300336	0.294113
RPS27A	3377	4145	2529	3496	4226	3003	1673	1383	1033	1231	1554	1447	0.709266	0.724788	0.702987	0.012829	0.044061
NOG2	36807	34881	17084	40466	26939	32813	628	811	472	566	579	548	0.980251	0.97966	0.981266	-0.00149	-0.00236
UBC5	135	102	93	201	95	108	467	380	710	743	575	375	0.217935	0.16437	0.182697	0.254449	-0.15251
DTD1	76	92	74	72	98	74	1073	1619	1525	2532	1907	1695	0.059957	0.036964	0.045355	0.402679	-0.29512
EPT1	201	137	189	248	242	176	609	688	616	815	687	653	0.207104	0.234042	0.2364	-0.19087	-0.01446
YJR079W	2493	2507	2366	2968	2032	1733	0	0	0	0	0	0	1	1	1	0	0
YJR112W-A	1584	1259	1039	1962	978	978	0	0	0	0	0	0	1	1	1	0	0
NSP1	362	222	264	309	72	106	347	274	433	367	226	203	0.479079	0.417933	0.292326	0.712685	0.515693
TEF4	4935	5006	1938	3104	2674	2739	47333	44246	25571	34239	36427	31970	0.098029	0.076786	0.07365	0.412519	0.060146
TUB3	457	405	206	330	359	245	2988	2534	2242	2908	2098	1746	0.135229	0.093033	0.134583	0.006904	-0.53269
TUB1	53	67	77	101	84	76	1707	1365	1382	2054	1463	1266	0.038451	0.049822	0.055465	-0.52858	-0.15481
YDR210W-	27	25	33	63	13	8	414	398	352	435	58	152	0.060163	0.10611	0.116549	-0.95399	-0.13538

	premRNA_fast_A	premRNA_fast_B	premRNA_slow_A	premRNA_slow_B	premRNA_wt_A	premRNA_wt_B	mRNA_fast_A	mRNA_fast_B	mRNA_slow_A	mRNA_slow_B	mRNA_wt_A	mRNA_wt_B	premRNA ratio_fast	premRNA ratio_slow	premRNA ratio_wt	premRNAratio_fast/wt (log2)	premRNA ratio_slow/wt(log2)
YOP1	644	724	316	711	842	650	3453	3176	3583	5283	4577	4152	0.171415	0.099833	0.14537	0.237763	-0.54215
YLL067C	62	46	104	64	84	93	0	0	0	0	0	0	1	1	1	0	0
SRC1	113	189	124	207	108	138	391	723	688	767	435	588	0.215722	0.182618	0.194489	0.149483	-0.09086
NMD2	91	107	101	116	35	75	236	430	325	454	87	87	0.238771	0.220299	0.374924	-0.65097	-0.76714
AI2	43245	63639	232	352	51011	68457	260	359	1	2	408	506	0.994207	0.995029	0.992364	0.002677	0.00387
YML6	97	105	66	71	48	88	417	325	314	561	384	217	0.216451	0.143013	0.199818	0.115355	-0.48254
NBL1	260	165	250	278	455	356	4270	4217	3412	3951	3404	2919	0.047525	0.067003	0.113304	-1.25346	-0.75791
YDR210C-D	12	31	91	197	69	45	259	359	900	1152	213	279	0.061884	0.11893	0.191785	-1.63185	-0.68937
ECM33	1602	1580	1733	2277	1994	1986	4519	4135	4905	7384	6026	5518	0.269094	0.248381	0.256644	0.068342	-0.04721
YSF3	598	550	1112	1379	743	518	87	67	28	97	133	54	0.882201	0.95486	0.876884	0.008722	0.122904
UBC12	357	431	652	867	430	371	120	148	157	211	117	109	0.746407	0.8051	0.779511	-0.06261	0.046598
UBC13	585	446	511	646	503	487	959	978	1425	1934	1137	1273	0.346044	0.257167	0.291706	0.246441	-0.18181
YLR202C	247	337	218	255	273	298	0	0	0	0	0	0	1	1	1	0	0
YOR318C	1943	1865	1174	1146	1135	973	0	0	0	0	0	0	1	1	1	0	0
MRK1	914	1078	291	549	597	508	7	36	5	31	117	28	0.980042	0.96483	0.891948	0.135884	0.113315
RPL20B	2770	3180	1395	2299	2435	2448	5246	5543	3568	4954	5648	5055	0.355056	0.299026	0.31376	0.178388	-0.06939
SPO22	97	110	107	133	76	154	3	0	0	0	0	0	0.985	1	1	-0.0218	0
RPL20A	2508	3058	1629	2222	2077	2650	6677	7391	5268	7502	8265	6804	0.282857	0.232348	0.240568	0.233626	-0.05016
RPS16B	2875	2873	1517	2319	2851	2554	24775	24828	18867	24463	29972	26523	0.103846	0.080505	0.087348	0.249609	-0.1177
RPL17A	2039	2081	1257	1780	2187	1937	38087	34432	25028	30360	32567	28388	0.053904	0.051602	0.063401	-0.23412	-0.29708
RPL17B	874	852	885	1411	1581	1687	19269	19379	19905	23563	23309	20512	0.042752	0.049534	0.069757	-0.70636	-0.49393
RPS16A	2909	3290	2137	3259	2995	3108	9436	10175	9242	13327	13092	12440	0.23999	0.192147	0.193036	0.314101	-0.00666
RPL26A	2567	2830	1823	2712	3471	2603	16077	15053	15999	21242	23946	20881	0.147968	0.107753	0.118721	0.317711	-0.13984
RPL26B	2837	3932	2543	3538	2349	2725	32691	32373	29412	38293	41235	40092	0.094079	0.08208	0.058769	0.6788	0.481957
SEC14	662	529	582	781	577	731	1747	1327	1618	2032	1453	1565	0.279912	0.271092	0.301308	-0.10627	-0.15245
RPS7A	3545	3785	2635	3514	3450	3321	77457	76820	48960	68065	74992	65096	0.045361	0.050082	0.046261	-0.02835	0.114486
SEC17	244	323	367	330	372	288	915	1256	1106	1591	823	805	0.207543	0.210468	0.287396	-0.46963	-0.44944
GIM5	116	186	235	335	194	172	592	563	716	817	658	569	0.206086	0.268953	0.229909	-0.15781	0.226293
TAN1	134	193	323	332	300	234	267	339	969	1178	816	814	0.348473	0.234934	0.24605	0.502098	-0.0667
VPS75	358	403	204	430	396	427	1186	1551	905	1265	987	941	0.219054	0.218818	0.299234	-0.44999	-0.45154
RPL6A	1869	1736	941	1284	1294	1219	10228	9716	8809	12512	13318	11724	0.153045	0.094792	0.09137	0.744169	0.053043
RPS7B	2022	1952	1568	2366	3173	2610	27095	23675	21254	22776	30048	24798	0.072807	0.081406	0.09537	-0.38946	-0.2284
YPR063C	129	136	60	107	135	107	1347	1385	1243	1853	1349	1086	0.088407	0.05032	0.09033	-0.03105	-0.84408
BET4	140	63	104	188	193	173	86	149	59	90	54	102	0.458319	0.657148	0.705234	-0.62175	-0.10188
HAC1	9598	9551	8447	12580	12406	10877	0	0	2	3	20	0	1	0.999762	0.999195	0.001161	0.000819
SRB2	257	298	283	353	362	303	1528	1171	978	1191	1336	883	0.173418	0.226526	0.234336	-0.43432	-0.0489
YGR109W-	0	7	2	5	3	7	162	297	285	397	367	299	0.011513	0.009703	0.015492	-0.42824	-0.67498
POP8	105	160	131	191	104	105	486	610	930	1257	840	923	0.192729	0.127687	0.106155	0.860401	0.266445
HNT2	319	344	134	323	243	319	457	454	454	570	365	373	0.42108	0.294797	0.430327	-0.03134	-0.54571
ERV1	608	812	633	910	947	716	1946	1714	1423	1931	1702	1477	0.279757	0.314095	0.341993	-0.28979	-0.12277
HNT1	856	869	993	1201	1045	1102	12555	11518	7318	10407	9005	7432	0.066991	0.111472	0.116555	-0.79897	-0.06434

	premRNA_fast_A	premRNA_fast_B	premRNA_slow_A	premRNA_slow_B	premRNA_wt_A	premRNA_wt_B	mRNA_fast_A	mRNA_fast_B	mRNA_slow_A	mRNA_slow_B	mRNA_wt_A	mRNA_wt_B	premRNA ratio_fast	premRNA ratio_slow	premRNA ratio_wt	premRNAratio_fast/wt (log2)	premRNA ratio_slow/wt(log2)
YNL050C	105	192	187	216	108	163	78	123	182	189	139	132	0.591647	0.520054	0.494895	0.257616	0.07154
YBL005W-E	70	38	63	214	43	62	1415	782	3141	4265	1241	939	0.04674	0.033721	0.047714	-0.02975	-0.50076
RPS14A	5304	6061	1692	2339	2619	2663	31736	26432	22857	27777	29542	27726	0.164865	0.073295	0.084532	0.963708	-0.20579
YBR062C	248	280	365	530	236	320	409	598	390	658	309	322	0.34819	0.464786	0.465735	-0.41963	-0.00294
YML133C	577	625	417	596	433	613	0	0	0	0	0	0	1	1	1	0	0
YDR381C-A	323	289	442	567	280	285	334	208	283	225	86	147	0.536559	0.662782	0.712375	-0.4089	-0.1041
RPL18A	2560	2926	1466	2369	2847	2976	55533	55818	33450	49158	53738	48442	0.046938	0.043981	0.054096	-0.20476	-0.29864
RPL35A	3773	3798	3452	4739	4943	4026	6139	5734	6292	7094	8010	6430	0.389549	0.37738	0.383326	0.02323	-0.02256
RPL35B	3061	3662	2386	3629	3131	2753	7343	7159	5349	6672	7275	7138	0.316315	0.330382	0.289609	0.127255	0.190029
RPL18B	2398	2636	2222	3114	3300	3152	5431	5147	3943	5682	6333	6272	0.322492	0.357223	0.338519	-0.06997	0.07759
DBP2	823	911	454	820	748	773	2266	2390	879	1297	1721	1383	0.271203	0.363963	0.330745	-0.28635	0.13807
YPR202W	889	997	780	1287	1000	890	43	52	27	42	12	23	0.952146	0.96747	0.981475	-0.04377	-0.02074
YHL009W-I	0	0	0	10	0	0	126	110	121	191	51	43	0	0.024876	0		inf
TAF14	638	706	1019	1159	760	735	433	485	361	352	277	356	0.594242	0.752724	0.703289	-0.24307	0.098004
PFY1	1349	1259	1168	1834	1835	1527	6920	6062	6727	8500	8193	7303	0.167555	0.162707	0.17796	-0.08692	-0.12928
SAR1	218	180	299	355	237	243	4008	3755	4494	5745	4240	3367	0.048664	0.06029	0.060125	-0.3051	0.003943
DCN1	61	65	146	113	71	89	24	11	27	53	54	31	0.786455	0.762327	0.654833	0.264237	0.219282
PSP2	211	276	215	268	170	272	165	220	182	223	112	115	0.558811	0.543693	0.65284	-0.22437	-0.26394
PRE3	573	480	444	661	1159	1084	4173	3881	3669	5165	3687	3027	0.1154	0.110704	0.251425	-1.12348	-1.18342
MPT5	1251	1171	907	979	756	705	14	0	29	40	33	10	0.994466	0.964881	0.972094	0.032826	-0.01074
ECM9	55	162	122	218	122	138	323	505	793	941	575	569	0.194191	0.160713	0.185113	0.069064	-0.20392
YLR227W-E	69	43	280	276	84	101	74	63	205	331	101	94	0.444089	0.516007	0.486001	-0.13011	0.086431
YPR170W-I	319	445	215	267	174	245	10897	10770	7646	10082	9824	8662	0.03406	0.026575	0.022455	0.601055	0.24303
MCM21	109	115	163	178	102	106	253	357	350	443	326	252	0.272375	0.302187	0.267204	0.027652	0.177501
YCL002C	525	500	563	782	981	788	216	353	114	161	209	120	0.647334	0.830439	0.846106	-0.38633	-0.02696
TFC3	46	117	50	181	179	171	388	551	628	701	410	369	0.14057	0.139481	0.310286	-1.14231	-1.15353
RPL13A	1464	1385	1182	1479	1750	1545	3803	4081	3445	4377	4717	3886	0.265671	0.254009	0.277541	-0.06306	-0.12782
RPL13B	3017	3210	1613	2140	2257	2107	5910	6838	4582	7116	8128	6312	0.328715	0.245786	0.2338	0.491563	0.07213
RUB1	568	537	583	853	678	657	3787	4262	4413	4678	2846	3312	0.121162	0.135458	0.178964	-0.56274	-0.40183
YNL284C-B	0	1	6	21	1	6	287	427	1639	1886	285	230	0.001168	0.00733	0.01446	-3.62969	-0.98025
RPL40A	11578	11571	9516	12981	15202	11219	12834	13138	13110	17679	19349	15927	0.471283	0.421982	0.426635	0.143589	-0.01582
SCS22	273	139	322	459	341	212	214	407	480	515	220	218	0.407577	0.436374	0.550433	-0.4335	-0.335
RPL22A	6370	7535	3731	6295	6333	6789	11845	12027	11417	13655	17459	16174	0.367449	0.280921	0.280916	0.387405	2.71E-05
RPL22B	11564	13487	7976	12443	13820	12781	384	309	532	649	697	683	0.972731	0.943949	0.95063	0.033158	-0.01017
QCR9	390	452	226	381	606	493	741	894	693	1160	634	742	0.340319	0.246581	0.44395	-0.38351	-0.84834
YHL050C	1013	880	919	1744	1120	1151	0	0	0	0	0	0	1	1	1	0	0
RPL40B	1335	1314	810	1473	1725	1690	16250	16269	15322	19020	22130	19643	0.075324	0.061044	0.075766	-0.00844	-0.31169
BUD25	1712	1614	1158	1535	1032	971	0	0	0	0	0	0	1	1	1	0	0
RPL36A	3231	4022	2693	3789	3071	3282	8508	8131	9153	12329	10654	10768	0.303092	0.231206	0.228673	0.406467	0.015894
RPS4A	1222	1514	825	1253	1516	1494	24674	22558	21900	26360	29382	23970	0.055042	0.04084	0.053868	0.0311	-0.39943
RPS14B	3614	3997	2598	3947	6078	5713	3418	2511	1744	1988	4471	3473	0.564052	0.63169	0.599047	-0.08684	0.076548

	premRNA_fast_A	premRNA_fast_B	premRNA_slow_A	premRNA_slow_B	premRNA_wt_A	premRNA_wt_B	mRNA_fast_A	mRNA_fast_B	mRNA_slow_A	mRNA_slow_B	mRNA_wt_A	mRNA_wt_B	premRNA ratio_fast	premRNA ratio_slow	premRNA ratio_wt	premRNAratio_fast/wt (log2)	premRNA ratio_slow/wt(log2)
RPL36B	1373	1313	894	961	1365	1132	34145	31134	29370	36932	43261	35144	0.039561	0.02745	0.030896	0.356649	-0.17061
RPS30B	1416	1744	1087	2059	1452	1471	7751	7140	5288	8186	8001	7222	0.175388	0.185743	0.161409	0.119822	0.202584
RPS30A	3479	4608	1919	3777	2680	2778	23433	22750	17665	21855	21698	15351	0.148853	0.122672	0.131585	0.177894	-0.1012
YFR045W	117	48	105	164	67	47	83	97	120	146	69	56	0.458017	0.497849	0.474479	-0.05094	0.069366
BIG1	148	83	115	126	140	95	552	736	569	768	391	537	0.156386	0.154534	0.206985	-0.40442	-0.4216
RPL19B	7766	7450	7081	9380	9475	9517	6090	5295	5504	6763	6282	6120	0.572511	0.571855	0.60497	-0.07956	-0.08121
SFT1	431	437	343	499	440	432	445	562	714	839	467	502	0.464723	0.348724	0.473821	-0.02797	-0.44226
RPL19A	2941	3235	2065	2987	3608	3476	2903	2940	2668	3606	3721	2728	0.513569	0.444677	0.526287	-0.03529	-0.24309
QCR10	37	99	85	104	45	64	213	264	227	293	198	164	0.210364	0.2672	0.232943	-0.14709	0.197942
DID4	31	162	151	158	115	125	490	468	598	676	433	403	0.158322	0.195525	0.223298	-0.49611	-0.19162
RPL6B	1880	2029	1415	1676	2143	1619	18820	19719	15785	20311	26280	21313	0.092059	0.079247	0.072998	0.334688	0.118495
YOL047C	183	174	344	454	179	164	0	7	0	6	39	3	0.980663	0.993478	0.901568	0.12132	0.140051
YOL103W-I	0	14	2	2	10	4	40	35	68	118	38	91	0.142857	0.022619	0.125219	0.190116	-2.46885
MOB2	47	64	80	104	86	95	69	34	31	65	59	33	0.529117	0.668053	0.667645	-0.3355	0.00088
KEI1	148	167	165	240	171	148	548	461	597	637	562	396	0.239284	0.245098	0.252673	-0.07855	-0.04392
CMC2	65	84	99	142	97	138	482	622	1442	1659	552	643	0.118905	0.071545	0.163079	-0.45576	-1.18865
CMC4	176	127	217	222	220	129	76	82	175	183	134	87	0.653034	0.55086	0.609346	0.099898	-0.14558
YIL082W-A	2	11	8	5	4	13	559	532	275	659	418	346	0.011911	0.017899	0.022845	-0.93954	-0.35198
IMD4	3148	3598	1521	2360	2349	2220	8303	9440	5532	8894	7927	7556	0.275437	0.212678	0.227839	0.273706	-0.09934
GOT1	193	187	167	249	186	161	1119	1077	997	1651	1333	1385	0.147523	0.137262	0.113294	0.380867	0.276853
STO1	507	579	665	601	593	444	588	669	490	645	484	335	0.463478	0.529051	0.560283	-0.27365	-0.08275
ERV41	162	182	150	222	213	214	1303	1122	1394	1424	1309	1082	0.125075	0.116011	0.152535	-0.28635	-0.39488
YPR153W	163	172	452	462	310	297	388	500	401	316	430	281	0.275889	0.561862	0.46638	-0.75742	0.268711
YCL019W	2	0	0	3	0	0	28	113	167	413	60	63	0.033333	0.003606	0	inf	inf
YLL066C	76	65	76	56	54	64	2	0	0	0	0	0	0.987179	1	1	-0.01862	0
CNB1	239	152	232	394	126	127	1548	1626	1949	2571	1525	1511	0.109617	0.119628	0.076925	0.510932	0.637027
OSW2	82	81	66	77	63	97	0	0	0	0	0	0	1	1	1	0	0
SAC6	85	150	148	153	83	108	2022	1702	1955	2719	2056	1723	0.060668	0.061824	0.048894	0.311279	0.338527
SNC1	232	97	142	286	198	280	3344	2785	2578	3437	3106	2649	0.049267	0.064513	0.077762	-0.65843	-0.26947
EST3	43	33	18	17	70	35	1914	1763	1544	2137	2066	1891	0.020173	0.009708	0.025472	-0.33646	-1.39167
GLC7	584	498	400	692	272	456	1721	1912	3037	4242	1838	2254	0.230001	0.128316	0.148588	0.630322	-0.21162
YDL012C	385	414	523	490	409	421	1846	1649	1777	2001	1438	968	0.186623	0.21205	0.262268	-0.49091	-0.30664
GIM4	256	290	396	873	242	276	977	881	836	1525	1057	944	0.227638	0.342741	0.206263	0.142252	0.732631
RPL21A	9477	9451	4750	6720	7641	7104	19766	19305	14565	19650	22326	21020	0.32637	0.250379	0.253788	0.362883	-0.01951
YBR219C	961	1028	729	1039	1165	882	0	0	0	0	0	0	1	1	1	0	0
SMD2	27	84	59	38	73	71	52	83	161	116	83	47	0.422383	0.257468	0.534822	-0.34051	-1.05467
RPL21B	6573	6264	3360	5560	6075	5277	14176	12659	9617	13305	14382	13667	0.323906	0.276823	0.287761	0.170704	-0.05591
RPS13	32757	33391	13861	20174	25585	21921	12713	12960	10449	14866	17348	14839	0.720402	0.572959	0.596128	0.273179	-0.05719
RPP1B	3459	3348	2721	4154	4685	5164	61825	60268	45962	65371	62118	59455	0.052806	0.05782	0.075023	-0.50663	-0.37576
MUD1	31	67	45	89	65	60	46	72	81	96	36	52	0.442306	0.419112	0.589639	-0.41479	-0.4925
RPS23B	2643	2903	1728	2786	2817	2721	47937	48544	34209	46954	46921	45832	0.05434	0.052048	0.056339	-0.05212	-0.11431

RP genes

	premRNA_fast_A	premRNA_fast_B	premRNA_slow_A	premRNA_slow_B	premRNA_wt_A	premRNA_wt_B	mRNA_fast_A	mRNA_fast_B	mRNA_slow_A	mRNA_slow_B	mRNA_wt_A	mRNA_wt_B	premRNA ratio_fast	premRNA ratio_slow	premRNA ratio_wt	premRNAratio_fast/wt (log2)	premRNA ratio_slow/wt(log2)
RPL23B	6219	6117	3896	5868	6985	6069	38703	35364	33114	42844	49086	40374	0.142953	0.112866	0.127625	0.163622	-0.1773
RPL23A	2696	2928	2252	2403	2900	2658	24928	26625	23757	33011	29283	28051	0.098336	0.07722	0.088332	0.154786	-0.19396
RPS23A	2079	2254	1471	2387	2630	2454	23914	21271	18446	26058	23788	21631	0.087898	0.078886	0.100721	-0.19647	-0.35252
RPS9B	8394	9030	4237	5585	9919	8361	25405	24513	18985	22399	27688	24705	0.258779	0.191017	0.258306	0.002637	-0.43538
RPS9A	4568	4763	3262	4822	5716	6217	212	219	839	975	996	849	0.955845	0.813613	0.865728	0.142863	-0.08957
PMI40	137	98	79	143	117	128	2069	2112	1664	2429	2254	2086	0.053224	0.050461	0.05358	-0.00963	-0.08652
RPS4B	1363	1384	561	1058	1308	996	13148	13605	8297	13005	12833	11929	0.093132	0.069283	0.084778	0.135572	-0.2912
OM14	67	131	105	191	151	218	309	266	346	481	386	290	0.254083	0.258521	0.355163	-0.48318	-0.4582
RRT8	57	133	25	84	89	47	311	382	362	487	318	292	0.206572	0.105855	0.178658	0.209442	-0.75511
PHO85	186	100	154	171	209	181	249	234	553	408	158	192	0.363494	0.256579	0.527368	-0.53688	-1.03941
YHR097C	102	91	56	156	64	65	314	217	277	385	201	235	0.270323	0.228262	0.229088	0.238784	-0.00521
YSC84	157	184	110	249	187	227	587	654	443	723	278	368	0.215296	0.227544	0.391832	-0.86391	-0.78409
GCR1	2773	3145	4598	6509	3655	3973	0	0	0	0	0	0	1	1	1	0	0
YCL012C	45	22	34	25	9	73	612	599	527	561	485	457	0.05196	0.051634	0.077977	-0.58565	-0.59473
RPL28	74219	81440	37112	60086	61627	58861	64685	64190	53985	76206	72229	72968	0.546772	0.424126	0.453446	0.270007	-0.09644
RPL25	7090	7022	6273	8017	9714	9022	16401	15748	13049	16963	18551	17237	0.305103	0.322796	0.343627	-0.17155	-0.09022
SPO1	447	505	339	469	309	335	25	18	0	37	7	0	0.956309	0.963439	0.988924	-0.04838	-0.03767
VMA10	371	301	586	706	490	435	850	1084	1270	1364	1256	841	0.260589	0.328398	0.310775	-0.2541	0.079573
VPS29	56	123	113	269	131	176	773	855	954	1673	1045	1110	0.096659	0.122211	0.124127	-0.36083	-0.02244
YPR098C	100	243	140	247	65	119	1095	1209	1096	1592	941	926	0.125519	0.12379	0.089244	0.492076	0.472073
MMS2	84	74	80	145	99	69	216	384	980	1230	564	628	0.220786	0.090463	0.124158	0.830466	-0.45678
ABP140	5	25	3	22	26	7	542	786	832	1081	678	764	0.019983	0.011769	0.023005	-0.20317	-0.96696
YEL076C-A	603	509	519	747	478	550	0	0	0	0	0	0	1	1	1	0	0
YIP3	189	311	230	235	175	208	801	723	748	848	840	760	0.245841	0.226082	0.193645	0.344314	0.223431
YPL109C	124	245	122	285	123	207	182	245	306	314	268	148	0.452614	0.38042	0.448838	0.012087	-0.2386
OST5	181	156	207	295	264	261	1970	1642	1908	2677	2354	1990	0.085455	0.098566	0.108394	-0.34305	-0.13713
RPS0A	2248	2230	1165	1730	2037	1714	20993	18887	17594	23039	23527	20927	0.101164	0.065974	0.077693	0.38084	-0.23587
RPS0B	3436	3944	2107	2795	4120	2971	28071	25287	17741	25125	24649	24511	0.12199	0.103132	0.125658	-0.04274	-0.28501
APS3	109	142	132	379	174	326	464	425	557	686	408	481	0.220334	0.273725	0.351467	-0.6737	-0.36066
YDR261C-D	11	42	102	187	41	40	44	67	186	198	37	66	0.292661	0.41994	0.4515	-0.6255	-0.10454
YJL113W	0	1	0	0	0	0	62	28	171	91	61	30	0.017241	0	0	inf	
NCE101	714	629	689	812	454	391	797	768	1007	1386	804	731	0.461393	0.387838	0.354688	0.379446	0.128907
PCH2	1323	1359	753	1127	865	947	10	2	6	24	9	12	0.995514	0.985622	0.988595	0.010063	-0.00435
YPR010C-A	55	93	105	111	152	77	894	835	595	824	639	461	0.079086	0.134358	0.167642	-1.0839	-0.3193
RPL34B	2486	2989	1943	3279	2795	2566	18937	18047	20883	26308	27757	22643	0.129067	0.097974	0.096636	0.41748	0.019835
RPL34A	3608	3709	2601	3807	3104	3089	33161	25903	27787	30446	37319	30105	0.11169	0.098368	0.084923	0.395261	0.21203
LSM7	149	129	121	329	194	178	719	870	951	1109	652	823	0.150394	0.170832	0.203568	-0.43677	-0.25294
RPL43A	8885	8716	5671	7026	8053	7478	14611	14106	10330	13222	15620	12328	0.380031	0.350706	0.358869	0.082657	-0.0332
LSM2	543	584	455	798	564	476	1625	1947	1791	3046	2200	2406	0.2406	0.205089	0.184608	0.382175	0.15179
RPL43B	3053	3135	3584	4954	4634	3965	2577	2367	1921	2675	2807	2542	0.556033	0.650204	0.616055	-0.14789	0.077835
RPS24B	1499	1589	885	1749	2196	1859	4255	3174	2362	3619	3516	3342	0.297064	0.299189	0.370943	-0.32042	-0.31014

	premRNA_ fast_A	premRNA_ fast_B	premRNA_ slow_A	premRNA_ slow_B	premRNA_ wt_A	premRNA_ wt_B	mRNA_ fast_A	mRNA_ fast_B	mRNA_ slow_A	mRNA_ slow_B	mRNA_ wt_A	mRNA_ wt_B	premRNA ratio_fast	premRNA ratio_slow	premRNA ratio_wt	premRNAratio_fast/wt (log2)	premRNA ratio_slow/ wt(log2)
RPS24A	6335	7118	3099	4826	4982	4635	10687	10767	8193	10148	10496	9874	0.385076	0.298367	0.320667	0.26407	-0.10399
REC102	243	443	106	216	230	229	16	0	12	9	19	9	0.969112	0.929153	0.94294	0.039498	-0.02125
PCC1	107	105	81	245	175	119	1268	1133	1243	1540	1077	854	0.081316	0.099217	0.131039	-0.68838	-0.40135
REC107	1398	1669	947	1591	1331	1312	118	124	127	124	21	125	0.926503	0.904724	0.94874	-0.03422	-0.06854
NYV1	132	132	103	227	210	218	551	433	653	610	355	439	0.213447	0.203725	0.351746	-0.72066	-0.78791
CGI121	321	397	280	493	544	457	374	505	520	765	567	523	0.451002	0.370946	0.477988	-0.08384	-0.36576
YMR045C	14	17	26	50	13	15	1253	1493	3574	4335	1018	1079	0.011154	0.009312	0.01316	-0.23861	-0.49895
RPL2A	924	1092	394	844	1357	906	5394	5358	5890	7737	8290	6754	0.157776	0.080528	0.129471	0.285243	-0.68507
RPL2B	6472	5942	4684	7374	7938	8872	3186	2880	2739	3951	4998	3900	0.671831	0.641069	0.65414	0.038497	-0.02912
RIM1	462	482	439	727	522	518	3931	3459	3411	4763	3230	3254	0.113736	0.123224	0.138227	-0.28135	-0.16575
IST1	61	39	33	97	66	84	194	273	459	569	237	388	0.182108	0.106359	0.197894	-0.11993	-0.89578
RPL39	2012	2755	1653	2310	2128	1885	4961	5006	5275	7402	6875	5900	0.321761	0.238224	0.239249	0.427476	-0.0062
AIM11	126	110	106	123	110	135	449	391	708	754	394	303	0.219346	0.135236	0.263237	-0.26315	-0.96088
YBR255C-A	215	146	255	277	269	147	317	237	364	442	312	202	0.392668	0.398606	0.442099	-0.17106	-0.14941
RPL30	6540	7192	5352	8270	10648	9792	18610	17113	11145	14212	19069	14409	0.277973	0.346136	0.381462	-0.4566	-0.1402
OAZ1	6	3	7	1	0	5	346	559	292	520	363	350	0.011192	0.012665	0.007042	0.668329	0.846781
UBC4	487	516	781	631	631	836	6677	6154	6756	6786	6256	5168	0.07267	0.094348	0.115431	-0.6676	-0.29096
EFB1	3566	4324	2957	4451	3976	3729	39560	42117	39259	53137	53145	47410	0.087898	0.073667	0.071263	0.302676	0.047879
SEC27	575	808	511	676	595	565	3794	4260	4128	6007	4233	3812	0.14552	0.105653	0.126162	0.205948	-0.25594
RPL33B	1088	1187	804	1357	1434	1140	17533	16158	13490	17122	24484	21241	0.063432	0.064841	0.053132	0.255617	0.287321
PTC7	382	493	387	598	607	424	725	635	760	902	600	448	0.391067	0.368034	0.494569	-0.33876	-0.42633
RPL33A	4693	4812	3193	4110	5007	4094	37134	35248	29923	35680	43726	35567	0.11616	0.099855	0.102984	0.173691	-0.04451

	premRNA_fast_A	premRNA_fast_B	premRNA_slow_A	premRNA_slow_B	premRNA_wt_A	premRNA_wt_B	mRNA_fast_A	mRNA_fast_B	mRNA_slow_A	mRNA_slow_B	mRNA_wt_A	mRNA_wt_B	premRNA ratio_fast	premRNA ratio_slow	premRNA ratio_wt	premRNA ratio_fast/wt(log2)	premRNA ratio_slow/wt(log2)
RPL13A	1464	1385	1182	1479	1750	1545	3803	4081	3445	4377	4717	3886	0.265671	0.254009	0.277541	-0.06306	-0.12782
RPL13B	3017	3210	1613	2140	2257	2107	5910	6838	4582	7116	8128	6312	0.328715	0.245786	0.2338	0.491563	0.07213
RPL14A	4015	4266	3099	3925	4950	4539	70593	66837	46404	65645	70143	65670	0.056906	0.05951	0.065284	-0.19815	-0.1336
RPL14B	2369	2245	1641	2333	2949	2462	24791	23821	14367	19682	18894	18656	0.086676	0.104242	0.125796	-0.53739	-0.27115
RPL16A	2026	2146	1419	2041	2529	2198	17526	16226	17937	22324	24017	22443	0.110215	0.078539	0.092235	0.256934	-0.2319
RPL16B	1573	1746	1120	1453	1863	1484	28001	27449	26926	33026	36431	35372	0.056497	0.041038	0.044457	0.345744	-0.11546
RPL17A	2039	2081	1257	1780	2187	1937	38087	34432	25028	30360	32567	28388	0.053904	0.051602	0.063401	-0.23412	-0.29708
RPL17B	874	852	885	1411	1581	1687	19269	19379	19905	23563	23309	20512	0.042752	0.049534	0.069757	-0.70636	-0.49393
RPL18A	2560	2926	1466	2369	2847	2976	55533	55818	33450	49158	53738	48442	0.046938	0.043981	0.054096	-0.20476	-0.29864
RPL18B	2398	2636	2222	3114	3300	3152	5431	5147	3943	5682	6333	6272	0.322492	0.357223	0.338519	-0.06997	0.07759
RPL19A	2941	3235	2065	2987	3608	3476	2903	2940	2668	3606	3721	2728	0.513569	0.444677	0.526287	-0.03529	-0.24309
RPL19B	7766	7450	7081	9380	9475	9517	6090	5295	5504	6763	6282	6120	0.572511	0.571855	0.60497	-0.07956	-0.08121
RPL20A	2508	3058	1629	2222	2077	2650	6677	7391	5268	7502	8265	6804	0.282857	0.232348	0.240568	0.233626	-0.05016
RPL20B	2770	3180	1395	2299	2435	2448	5246	5543	3568	4954	5648	5055	0.355056	0.299026	0.31376	0.178388	-0.06939
RPL21A	9477	9451	4750	6720	7641	7104	19766	19305	14565	19650	22326	21020	0.32637	0.250379	0.253788	0.362883	-0.01951
RPL21B	6573	6264	3360	5560	6075	5277	14176	12659	9617	13305	14382	13667	0.323906	0.276823	0.287761	0.170704	-0.05591
RPL22A	6370	7535	3731	6295	6333	6789	11845	12027	11417	13655	17459	16174	0.367449	0.280921	0.280916	0.387405	2.71E-05
RPL22B	11564	13487	7976	12443	13820	12781	384	309	532	649	697	683	0.972731	0.943949	0.95063	0.033158	-0.01017
RPL23A	2696	2928	2252	2403	2900	2658	24928	26625	23757	33011	29283	28051	0.098336	0.07722	0.088332	0.154786	-0.19396
RPL23B	6219	6117	3896	5868	6985	6069	38703	35364	33114	42844	49086	40374	0.142953	0.112866	0.127625	0.163622	-0.1773
RPL25	7090	7022	6273	8017	9714	9022	16401	15748	13049	16963	18551	17237	0.305103	0.322796	0.343627	-0.17155	-0.09022
RPL26A	2567	2830	1823	2712	3471	2603	16077	15053	15999	21242	23946	20881	0.147968	0.107753	0.118721	0.317711	-0.13984
RPL26B	2837	3932	2543	3538	2349	2725	32691	32373	29412	38293	41235	40092	0.094079	0.08208	0.058769	0.6788	0.481957
RPL27A	3333	3037	2196	3149	2675	2548	32790	33478	24217	31250	34002	34096	0.08772	0.087342	0.071234	0.300336	0.294113
RPL27B	2098	2648	1497	2016	2164	2386	8300	8720	7633	10549	10009	10097	0.217352	0.162205	0.184455	0.236763	-0.18545
RPL28	74219	81440	37112	60086	61627	58861	64685	64190	53985	76206	72229	72968	0.546772	0.424126	0.453446	0.270007	-0.09644
RPL2A	924	1092	394	844	1357	906	5394	5358	5890	7737	8290	6754	0.157776	0.080528	0.129471	0.285243	-0.68507
RPL2B	6472	5942	4684	7374	7938	8872	3186	2880	2739	3951	4998	3900	0.671831	0.641069	0.65414	0.038497	-0.02912
RPL30	6540	7192	5352	8270	10648	9792	18610	17113	11145	14212	19069	14409	0.277973	0.346136	0.381462	-0.4566	-0.1402
RPL31A	2001	2007	1469	2027	1901	1935	61377	58478	41713	51267	58439	50652	0.032377	0.036027	0.03415	-0.07693	0.077154
RPL31B	947	1042	993	1240	1704	1264	9305	9366	11632	15559	13204	13276	0.096244	0.076234	0.100617	-0.06411	-0.40037
RPL33A	4693	4812	3193	4110	5007	4094	37134	35248	29923	35680	43726	35567	0.11616	0.099855	0.102984	0.173691	-0.04451
RPL33B	1088	1187	804	1357	1434	1140	17533	16158	13490	17122	24484	21241	0.063432	0.064841	0.053132	0.255617	0.287321
RPL34A	3608	3709	2601	3807	3104	3089	33161	25903	27787	30446	37319	30105	0.11169	0.098368	0.084923	0.395261	0.21203
RPL34B	2486	2989	1943	3279	2795	2566	18937	18047	20883	26308	27757	22643	0.129067	0.097974	0.096636	0.41748	0.019835
RPL35A	3773	3798	3452	4739	4943	4026	6139	5734	6292	7094	8010	6430	0.389549	0.37738	0.383326	0.02323	-0.02256
RPL35B	3061	3662	2386	3629	3131	2753	7343	7159	5349	6672	7275	7138	0.316315	0.330382	0.289609	0.127255	0.190029
RPL36A	3231	4022	2693	3789	3071	3282	8508	8131	9153	12329	10654	10768	0.303092	0.231206	0.228673	0.406467	0.015894
RPL36B	1373	1313	894	961	1365	1132	34145	31134	29370	36932	43261	35144	0.039561	0.02745	0.030896	0.356649	-0.17061
RPL37A	3353	3119	2428	3464	3589	3342	7605	7058	6854	7806	8238	7791	0.306231	0.284473	0.301823	0.020915	-0.08541
RPL37B	2912	3477	1721	2196	1860	1567	13952	11459	8860	10506	11712	9786	0.202734	0.167768	0.137536	0.559781	0.286659

	premRNA_fast_A	premRNA_fast_B	premRNA_slow_A	premRNA_slow_B	premRNA_wt_A	premRNA_wt_B	mRNA_fast_A	mRNA_fast_B	mRNA_slow_A	mRNA_slow_B	mRNA_wt_A	mRNA_wt_B	premRNA ratio_fast	premRNA ratio_slow	premRNA ratio_wt	premRNA ratio_fast/wt(log2)	premRNA ratio_slow/wt(log2)
RPL39	2012	2755	1653	2310	2128	1885	4961	5006	5275	7402	6875	5900	0.321761	0.238224	0.239249	0.427476	-0.0062
RPL40A	11578	11571	9516	12981	15202	11219	12834	13138	13110	17679	19349	15927	0.471283	0.421982	0.426635	0.143589	-0.01582
RPL40B	1335	1314	810	1473	1725	1690	16250	16269	15322	19020	22130	19643	0.075324	0.061044	0.075766	-0.00844	-0.31169
RPL42A	11151	11837	12080	16110	16205	16179	5470	4413	4676	5711	6610	5455	0.699665	0.729608	0.729064	-0.05938	0.001075
RPL42B	2253	2601	1548	2454	2372	2549	14555	13001	8923	11540	16536	15128	0.150376	0.161599	0.134824	0.157499	0.261339
RPL43A	8885	8716	5671	7026	8053	7478	14611	14106	10330	13222	15620	12328	0.380031	0.350706	0.358869	0.082657	-0.0332
**RPL43B**	3053	3135	3584	4954	4634	3965	2577	2367	1921	2675	2807	2542	0.556033	0.650204	0.616055	-0.14789	0.077835
RPL6A	1869	1736	941	1284	1294	1219	10228	9716	8809	12512	13318	11724	0.153045	0.094792	0.09137	0.744169	0.053043
RPL6B	1880	2029	1415	1676	2143	1619	18820	19719	15785	20311	26280	21313	0.092059	0.079247	0.072998	0.334688	0.118495
RPO26	388	518	656	800	774	761	1785	1731	2349	2777	2945	2348	0.20444	0.220977	0.226447	-0.1475	-0.03528
RPP1B	3459	3348	2721	4154	4685	5164	61825	60268	45962	65371	62118	59455	0.052806	0.05782	0.075023	-0.50663	-0.37576
RPS0A	2248	2230	1165	1730	2037	1714	20993	18887	17594	23039	23527	20927	0.101164	0.065974	0.077693	0.38084	-0.23587
RPS0B	3436	3944	2107	2795	4120	2971	28071	25287	17741	25125	24649	24511	0.12199	0.103132	0.125658	-0.04274	-0.28501
RPS10A	2958	3108	2017	3432	3742	3886	39842	37670	35960	43875	45573	40250	0.072665	0.062829	0.081963	-0.17371	-0.38353
RPS10B	2235	2413	1848	2647	2051	2076	30938	27425	24684	28147	24466	21717	0.074122	0.077805	0.0823	-0.15098	-0.08102
RPS11A	2284	2349	1378	2189	2451	2033	44844	40867	41816	49592	60786	47667	0.051409	0.037088	0.039832	0.368095	-0.10297
RPS11B	2947	3426	1945	2912	3469	2996	23216	21589	19059	23283	29458	25085	0.124799	0.101884	0.106023	0.235231	-0.05745
RPS13	32757	33391	13861	20174	25585	21921	12713	12960	10449	14866	17348	14839	0.720402	0.572959	0.596128	0.273179	-0.05719
RPS14A	5304	6061	1692	2339	2619	2663	31736	26432	22857	27777	29542	27726	0.164865	0.073295	0.084532	0.963708	-0.20579
**RPS14B**	3614	3997	2598	3947	6078	5713	3418	2511	1744	1988	4471	3473	0.564052	0.63169	0.599047	-0.08684	0.076548
RPS16A	2909	3290	2137	3259	2995	3108	9436	10175	9242	13327	13092	12440	0.23999	0.192147	0.193036	0.314101	-0.00666
RPS16B	2875	2873	1517	2319	2851	2554	24775	24828	18867	24463	29972	26523	0.103846	0.080505	0.087348	0.249609	-0.1177
RPS17A	8545	7274	6069	7438	9703	8882	17861	17134	15351	20995	26661	21157	0.310809	0.272465	0.281256	0.144144	-0.04581
RPS17B	2307	2659	1159	1809	2513	2020	4141	4492	4621	5765	5737	4407	0.364811	0.219681	0.309453	0.23743	-0.49431
RPS18A	2935	2986	2103	3215	3425	3251	29420	29306	23611	33687	30949	33262	0.091591	0.084453	0.094338	-0.04264	-0.15968
RPS18B	1113	1204	971	1151	1045	1252	23529	24150	21514	31723	30896	32434	0.046327	0.039098	0.034942	0.40691	0.162161
**RPS19A**	2397	2393	1629	2372	2452	2225	16794	16373	13905	17881	15652	15929	0.12621	0.110993	0.129001	-0.03156	-0.21692
RPS19B	2845	2911	1683	2603	2719	2406	25124	25245	22186	28165	27950	25344	0.102554	0.077555	0.08768	0.226072	-0.17701
RPS21A	2681	2194	1338	2088	2601	2657	31967	32994	19657	27747	29812	27827	0.069865	0.066857	0.083703	-0.26072	-0.3242
**RPS21B**	886	935	744	1058	1233	1217	28754	29480	20819	30790	31625	28061	0.030317	0.033862	0.039546	-0.38342	-0.22387
RPS22B	4582	4571	2017	3779	5328	5360	14320	13836	8298	9625	11326	9231	0.245369	0.238736	0.343636	-0.48593	-0.52547
RPS23A	2079	2254	1471	2387	2630	2454	23914	21271	18446	26058	23788	21631	0.087898	0.078886	0.100721	-0.19647	-0.35252
RPS23B	2643	2903	1728	2786	2817	2721	47937	48544	34209	46954	46921	45832	0.05434	0.052048	0.056339	-0.05212	-0.11431
RPS24A	6335	7118	3099	4826	4982	4635	10687	10767	8193	10148	10496	9874	0.385076	0.298367	0.320667	0.26407	-0.10399
RPS24B	1499	1589	885	1749	2196	1859	4255	3174	2362	3619	3516	3342	0.297064	0.299189	0.370943	-0.32042	-0.31014
RPS27A	3377	4145	2529	3496	4226	3003	1673	1383	1033	1231	1554	1447	0.709266	0.724788	0.702987	0.012829	0.044061
RPS27B	1964	2719	1359	2193	2391	1937	16178	15957	15716	23960	24736	24605	0.126923	0.081721	0.08056	0.655815	0.020653
RPS30A	3479	4608	1919	3777	2680	2778	23433	22750	17665	21855	21698	15351	0.148853	0.122672	0.131585	0.177894	-0.1012
RPS30B	1416	1744	1087	2059	1452	1471	7751	7140	5288	8186	8001	7222	0.175388	0.185743	0.161409	0.119822	0.202584
RPS4A	1222	1514	825	1253	1516	1494	24674	22558	21900	26360	29382	23970	0.055042	0.04084	0.053868	0.0311	-0.39943
RPS4B	1363	1384	561	1058	1308	996	13148	13605	8297	13005	12833	11929	0.093132	0.069283	0.084778	0.135572	-0.2912

	premRNA_fast_A	premRNA_fast_B	premRNA_slow_A	premRNA_slow_B	premRNA_wt_A	premRNA_wt_B	mRNA_fast_A	mRNA_fast_B	mRNA_slow_A	mRNA_slow_B	mRNA_wt_A	mRNA_wt_B	premRNA ratio_fast	premRNA ratio_slow	premRNA ratio_wt	premRNA ratio_fast/wt(log2)	premRNA ratio_slow/wt(log2)
RPS6A	1627	1788	1264	2138	1999	1861	6254	6308	6391	9254	8148	8710	0.213648	0.176398	0.186526	0.195859	-0.08054
RPS6B	4319	4714	2009	3067	3773	3055	13989	16458	11840	18193	17903	16307	0.22928	0.144663	0.165923	0.466594	-0.19782
RPS7A	3545	3785	2635	3514	3450	3321	77457	76820	48960	68065	74992	65096	0.045361	0.050082	0.046261	-0.02835	0.114486
RPS7B	2022	1952	1568	2366	3173	2610	27095	23675	21254	22776	30048	24798	0.072807	0.081406	0.09537	-0.38946	-0.2284
RPS9A	4568	4763	3262	4822	5716	6217	212	219	839	975	996	849	0.955845	0.813613	0.865728	0.142863	-0.08957
RPS9B	8394	9030	4237	5585	9919	8361	25405	24513	18985	22399	27688	24705	0.258779	0.191017	0.258306	0.002637	-0.43538

Table S2. Genes with non-canonical 5' splice sites and their splicing efficiency

Of 24 RPs (including YML6*, a mitochondrial RP) with non-canonical 5'SS, 19 splice worse (red) and 5 splice better (green) with fast RNAPII
Of 12 non-RPs with the same non-canonical 5'SS, 4 splice worse and 8 splice better with fast RNAPII The non-RPs are colour coded in the 1st column. The RPs are colour coded in the 2nd column. Non-consensus Branch Point sequences are also colored red. **5'SS GTACGT**: 19 introns: (including
14 RPs, plus YML6*, a mitochondrial RP)

ORF	Aliases	position	5' SS	Branch Point	3' SS
YBR189W	RPS9B	chrII:604509-604932	GTACGT	T**GACTAAC**AAGA	TTTTAG
YDL012C		chrIV:431467-431391	GTACGT	**GTACTAAC**GAGC	ATTCAG
YDR447C	RPS17B	chrIV:1355545-1355241	GTACGT	**TTACTAAC**TTAA	TTATAG
YDR471W	RPL27B	chrIV:1401795-1402189	GTACGT	**TTACTAAC**AAAT	TCGTAG
YFL034C-A	RPL22B	chrVI:64915-64604	GTACGT	**TTACTAAC**ATAA	TAACAG
YGL226C-A	OST5	chrVII:73132-72993	GTACGT	**TTACTAAC**AAGT	CTTAAG
YHR010W	RPL27A	chrVIII:126546-127117	GTACGT	**TTACTAAC**TATT	ACACAG
YIL133C	RPL16A	chrIX:99380-99100	GTACGT	**ATACTAAC**AAAT	AACCAG
YJL136C	RPS21B	chrX:157244-156794	GTACGT	**GTACTAAC**ATTG	TACTAG
YJL191W	RPS14B	chrX:73791-74209	GTACGT	**TTACTAAC**AACT	GTATAG
YLR287C-A	RPS30A	chrXII:713150-712730	GTACGT	**ATACTAAC**ATGG	ATTTAG
YLR306W	UBC12	chrXII:744148-744292	GTACGT	**ATACTAAC**ATAA	AAATAG
YML025C	YML6*	chrXIII:225333-225244	GTACGT	**ATACTAAC**ATTA	CAATAG
YMR033W	ARP9	chrXIII:337812-337908	GTACGT	**ATACTAAC**AACA	TAACAG
YNL069C	RPL16B	chrXIV:494968-494529	GTACGT	**TTACTAAC**TTTA	TTATAG
YNL302C	RPS19B	chrXIV:62918-62377	GTACGT	**TTACTAAC**AAAA	AATTAG
YOL121C	RPS19A	chrXV:92825-92445	GTACGT	**TTACTAAC**AATA	CTACAG
YOR182C	RPS30B	chrXV:678785-678384	GTACGT	**TTACTAAC**ATTA	CCTTAG
YPL081W	RPS9A	chrXVI:404951-405462	GTACGT	T**CACTAAC**AATG	AAACAG

5'SS GTATGC: 8 introns, including **2 RPs**

ORF	Aliases	position	5' SS	Branch Point	3' SS
YBL059W		chrII:110874-110953	GTATCG	**TTACTAAC**ATAA	ACATAG
YCL002C		chrIII:111628-111562	GTATGC	TT**GACTAAC**TAA	ACATAG
YDR305C	HNT2	chrIV:1073396-1073317	GTATGC	**TTACTAAC**TATA	TTACAG
YEL003W	GIM4 PFD2	chrV:148189-148287	GTATGC	CT**TACTAAC**AAT	GTATAG
YIL148W	RPL40A	chrIX:68710-69154	GTATGC	**TTACTAAC**TTGT	TAACAG
YJR094W-A	RPL43B	chrX:608301-608586	GTATGC	**TTACTAAC**AAAT	TTGCAG
YLL050C	COF1	chrXII:40395-40226	GTATGC	**TTACTAAC**TAAT	GTGTAG
YNL050C	YNL050C	chrXIV:534960-534879	GTATGC	**TTACTAAC**TTTT	TTGCAG

5’SS GTATGA: 6 introns in our data (**5 RPs**)

ORF	Aliases	position	5’ SS	Branch Point	3’ SS
YBR048W	RPS11B	chrII:332870-333391	GTATGA	**TTACTAAC**ATGC	ATACAG
YDR025W	RPS11A	chrIV:491554-491903	GTATGA	**TTACTAAC**CTTT	TTTTAG
YIL004C	BET1	chrIX:348489-348368	GTATGA	**TTACTAAC**TATA	ATATAG
YLR061W	RPL22A	chrXII:263200-263599	GTATGA	**TTACTAAC**ATTA	ATATAG
YPL090C	RPS6A	chrXVI:378384-378000	GTATGA	**ATACTAAC**AAAT	AAATAG
YPR043W	RPL43A	chrXVI:654162-654575	GTATGA	**TTACTAAC**ATAT	ATACAG

5’SS GTCNNN: **1 RP**

ORF	Aliases	position	5’ SS	Branch Point	3’ SS
YGL030W	RPL30	chrVII:439088-439328	GTCAGT	C**TACTAAC**AAGT	CAACAG

5’SS GTGNNN: 2 introns, including **1 RPG**

ORF	Aliases	position	5’ SS	Branch Point	3’ SS
YML034W	SRC1	chrXIII:211439-211575	GTGAGT	**TTACTAAC**ATCT	CTCTAG
YMR242C	RPL20A	chrXIII:754214-753747	GTGAGT	CA**TACTAAC**ATT	TATCAG

The following non-canonical 5’SS are not found in RPs

5’SS GTAGTA: 1 intron – **no RPs**

ORF	Aliases	position	5’ SS	Branch Point	3’ SS
YCL012C		chrIII:101695-101638	GTAGTA	**ATACTAAC**AGCT	TAATAG

5’SS GTAANN: 11 introns – **no RPs.** **N.B. GTAAGT stabilises interaction with U1 snRNA**
Of 11 for which we have data (but low counts, so not all reliable), 3 splice worse and 8 splice better with fast RNAPII. Some also have non-consensus BP sequences (red)

ORF	Aliases	position	5’ SS	Branch Point	3’SS
YAL030W	SNC1	chrI:87382-87505	GTAAGT	**ATACTAAC**TTTC	TTTTAG
YBL059C-A		chrII:110500-110425	GTAAGT	**TTACTAAC**ATTG	TTTTAG
YDL064W	UBC9	chrIV:337519-337639	GTAAGT	**ATACTAAC**AAAT	TAACAG
YFR045W		chrVI:242004-242086	GTAAGT	TT**TATTAAC**GCT	TTGCAG
YHR001W-A	QCR10	chrVIII:107826-107899	GTAAGT	**ATACTAAC**ATTT	ATACAG
YIL156W-A	YIL156W-B	chrIX:47693-47765	GTAAGT	A**ATACTAAC**AAG	GGTTAG
YKR095W-A	PCC1	chrXI:625896-625981	GTAAGT	**TTACTAAC**TATT	CATTAG
YLR211C		chrXII:564508-564459	GTAAGT	T**GACTAAC**ATGA	AATTAG
YLR445W		chrXII:1024567-1024659	GTAAGT	TT**TACTAAT**AAT	TAACAG
YNL012W	SPO1	chrXIV:609785-609879	GTAAGT	A**AACTAAC**CGAA	TATTAG
YNL044W	YIP3	chrXIV:545286-545375	GTAAGT	**ATACTAAC**GCGT	CTATAG
